# Cannabis use and cognitive biases in people with first-episode psychosis and their siblings

**DOI:** 10.1017/S0033291724001715

**Published:** 2024-11

**Authors:** L. Roldan, T. Sánchez-Gutiérrez, I. Fernández-Arias, E. Rodríguez-Toscano, G. López, J. Merchán-Naranjo, A. Calvo, M. Rapado-Castro, M. Parellada, C. Moreno, L. Ferraro, D. La Barbera, C. La Cascia, G. Tripoli, M. Di Forti, R.M. Murray, D. Quattrone, C. Morgan, C. Gayer-Anderson, P. B. Jones, H.E. Jongsma, J.B. Kirkbride, J. van Os, P. García-Portilla, S. Al-Halabí, J. Bobes, L. de Haan, M. Bernardo, J.L. Santos, J. Sanjuán, M. Arrojo, A. Szoke, B.P. Rutten, S. A. Stilo, I. Tarricone, A. Lasalvia, S. Tosato, P.-M. Llorca, P. Rossi Menezes, J-P Selten, A. Tortelli, E. Velthorst, C.M. Del-Ben, C. Arango, C. M. Díaz-Caneja

**Affiliations:** 1 Department of Personality, Assessment and Clinical Psychology, School of Psychology, Universidad Complutense de Madrid, Spain; 2 Department of Psychology, University of Córdoba, Spain; 3 Department of Experimental Psychology, Cognitive processes and Speech therapy, School of Psychology, Universidad Complutense de Madrid (UCM); Madrid, Spain; 4 Department of Child and Adolescent Psychiatry, Institute of Psychiatry and Mental Health, Hospital General Universitario Gregorio Marañón, IiSGM, CIBERSAM, School of Medicine, Universidad Complutense, Madrid, Spain; 5 Melbourne Neuropsychiatry Centre, Department of Psychiatry, The University of Melbourne and Melbourne Health, 161 Barry Street, Carlton South, Victoria 3053, Australia; 6 Department of Biomedicine, Neuroscience and Advanced Diagnostics (BiND), Psychiatry Section, University of Palermo, Palermo, Italy; 7 Social, Genetic, and Developmental Psychiatry Centre, Institute of Psychiatry, Psychology and Neuroscience, King's College London, London, England; 8 ESRC Centre for Society and Mental Health, Institute of Psychiatry, Psychology & Neuroscience, King's College London, London, UK; 9 Department of Psychosis Studies, Institute of Psychiatry, Psychology and Neuroscience, King's College London, London, UK; 10 Department of Health Services and Population Research, Institute of Psychiatry, Psychology and Neuroscience, King's College London, England; 11 Department of Psychiatry, University of Cambridge; 24CAMEO Early Intervention Service, Cambridgeshire & Peterborough NHS Foundation Trust, Cambridge, CB21 5EF, UK; 12 Psylife Group, Division of Psychiatry, University College London, 6th Floor, Maple House, 149 Tottenham Court Road, London W1T 7NF, UK; 13 Department of Psychiatry and Neuropsychology, School for Mental Health and Neuroscience, Maastricht University Medical Center, Maastricht, Netherlands; 14 Department Psychiatry, Brain Centre Rudolf Magnus, Utrecht University Medical Centre, Utrecht, The Netherlands; 15 Department of Medicine -Psychiatry, Universidad de Oviedo, ISPA, INEUROPA, CIBERSAM, Oviedo, Spain; 16 Department of Psychology, University of Oviedo, Spain; 17 Amsterdam UMC, University of Amsterdam, Academic Psychiatric Centre, Early Psychosis Department, Arkin, Amsterdam, The Netherlands; 18 Barcelona Clinic Schizophrenia Unit, Hospital Clinic, Departament de Medicina, Institut de Neurociències (UBNeuro), Universitat de Barcelona (UB), Institut d'Investigacions Biomèdiques August Pi I Sunyer (IDIBAPS), CIBERSAM, ISCIII, Barcelona, Spain; 19 Department of Psychiatry, Servicio de Psiquiatría Hospital “Virgen de la Luz,” Cuenca, Spain; 20 Department of Psychiatry, Hospital Clínico Universitario de Valencia, INCLIVA, CIBERSAM, School of Medicine, Universidad de Valencia, Valencia, Spain; 21 Department of Psychiatry, Psychiatric Genetic Group, Instituto de Investigación Sanitaria de Santiago de Compostela, Complejo Hospitalario Universitario de Santiago de Compostela, Santiago de Compostela, Spain; 22 Univ Paris Est Creteil, INSERM, IMRB, Fondation FondaMental, F-94010 Creteil, France; 23 AP-HP, Hopitaux Universitaires “ H. Mondor ”, DMU IMPACT, F-94010 Creteil, France; 24 Department of Mental Health and Addiction Services, ASP Crotone, Crotone, Italy; 25 Bologna Transcultural Psychosomatic Team (BoTPT), Department of Medical and Surgical Sciences, Alma Mater Studiorum University of Bologna; 26 Section of Psychiatry, Azienda Ospedaliera Universitaria Integrata di Verona, Verona, Italy; 27 Université Clermont Auvergne, EA 7280, Clermont-Ferrand 63000, France; 28 Department of Preventive Medicine, Faculdade de Medicina, Universidade of São Paulo, São Paulo, Brazil; 29 Etablissement Public de Santé Maison Blanche, Paris 75020, France; 30 Mental Health Service Organization ‘GGZ Noord-Holland-Noord’, Department of Research, the Netherlands; 31 Division of Psychiatry, Department of Neuroscience and Behavior, Ribeirão Preto Medical School, University of São Paulo, São Paulo, Brazil

**Keywords:** aberrance salience, cannabis, cognition, facial recognition, jumping to conclusions

## Abstract

**Background:**

Cannabis use and familial vulnerability to psychosis have been associated with social cognition deficits. This study examined the potential relationship between cannabis use and cognitive biases underlying social cognition and functioning in patients with first episode psychosis (FEP), their siblings, and controls.

**Methods:**

We analyzed a sample of 543 participants with FEP, 203 siblings, and 1168 controls from the EU-GEI study using a correlational design. We used logistic regression analyses to examine the influence of clinical group, lifetime cannabis use frequency, and potency of cannabis use on cognitive biases, accounting for demographic and cognitive variables.

**Results:**

FEP patients showed increased odds of facial recognition processing (FRP) deficits (OR = 1.642, CI 1.123–2.402) relative to controls but not of speech illusions (SI) or jumping to conclusions (JTC) bias, with no statistically significant differences relative to siblings. Daily and occasional lifetime cannabis use were associated with decreased odds of SI (OR = 0.605, CI 0.368–0.997 and OR = 0.646, CI 0.457–0.913 respectively) and JTC bias (OR = 0.625, CI 0.422–0.925 and OR = 0.602, CI 0.460–0.787 respectively) compared with lifetime abstinence, but not with FRP deficits, in the whole sample. Within the cannabis user group, low-potency cannabis use was associated with increased odds of SI (OR = 1.829, CI 1.297–2.578, FRP deficits (OR = 1.393, CI 1.031–1.882, and JTC (OR = 1.661, CI 1.271–2.171) relative to high-potency cannabis use, with comparable effects in the three clinical groups.

**Conclusions:**

Our findings suggest increased odds of cognitive biases in FEP patients who have never used cannabis and in low-potency users. Future studies should elucidate this association and its potential implications.

## Background

Cannabis use can increase the risk of psychosis (Di Forti et al., 2015; Di Forti et al., 2019; Karpov, Lindgren, Kieseppa, Wegelius, & Suvisaari, 2021; Luzi, Morrison, Powell, di Forti, & Murray, 2008; Murray, Paparelli, Morrison, Marconi, & Di Forti, 2013) and its prevalence is higher in patients diagnosed with schizophrenia (Bersani, Orlandi, Kotzalidis, & Pancheri, 2002; Hartz et al., 2014), bipolar disorder, depressive and anxiety disorders, and post-traumatic stress disorder (PTSD) (Koenfal, Gabrys, & Porath, 2019; Lev-Ran, Le Foll, McKenzie, George, & Rehm, 2013) than in healthy controls.

Some cognitive and reasoning biases have been reported across the psychosis continuum including people with first episode psychosis (FEP), chronic psychosis, clinical and familial high risk for psychosis, and non-clinical samples with psychotic-like experiences (Henquet et al., 2022; Langdon, Still, Connors, Ward, & Catts, 2014; Linney, Peters, & Ayton, 1998; Moritz, Van Quaquebeke, & Lincoln, 2012; Van Dael et al., 2006). These cognitive biases could increase psychosis risk by affecting the perception of others, decision-making about social situations, the meaning attributed to certain social stimuli, and real-life functioning (Green, Horan, & Lee, 2019; Mucci et al., 2021).

Aberrant salience is the unusual or incorrect assignment of meaning to neutral stimuli, which can favor the development of attentional biases that may in turn lead to the perception that the environment is dangerous and to the development of paranoid ideation (Howes & Murray, 2014; Kapur, 2003). Experimental illusion studies have focused on the paradigm of hearing voices in neutral random signals (white noise) in the absence of actual speech (speech illusion, SI) (Galdos et al., 2011).

Other cognitive biases have also consistently been reported in patients with schizophrenia (Hofer, Biedermann, Yalcin, & Fleischhacker, 2010; Lee et al., 2013), such as the jumping to conclusion (JTC) reasoning bias: under conditions of uncertainty, people with delusions use less information to arrive at a decision and express greater confidence in their judgment than controls (Dudley, Taylor, Wickham, & Hutton, 2016; Garety & Freeman, 2013; Lincoln, Ziegler, Mehl, & Rief, 2010; Murray et al., 2020; So, Siu, Wong, Chan, & Garety, 2016; Tripoli et al., 2021).

Also, deficits in facial recognition processing (discrimination, encoding, and recognition) have been found in people with schizophrenia (Archer, Hay, & Young, 1992; Shin et al., 2008; van 't Wout, Aleman, Kessels, Laroi, & Kahn, 2004; Walther et al., 2009), with milder degrees of facial recognition impairments observed in non-affected first-degree relatives of patients with schizophrenia (Li, Chan, Zhao, Hong, & Gong, 2010), thus suggesting that this could be a potential endophenotypic marker of psychosis (Fusar-Poli et al., 2022a; Soria Bauser et al., 2012; Tripoli et al., 2022).

Despite consistent evidence supporting the presence of impairments across cognition and social cognition in schizophrenia (Green et al., 2019; Velthorst et al., 2017), the effect of cannabis use on cognitive and social cognitive functioning in patients with FEP and their siblings is still controversial (Arnold, Allott, Farhall, Killackey, & Cotton, 2015; Bruins, Pijnenborg, investigators, Visser, & Castelein, 2021; Clausen et al., 2014; Meijer et al., 2012). Most studies report better or similar neuropsychological functioning in people with schizophrenia and FEP that use cannabis than in those who do not (Rabin, Zakzanis, & George, 2011; Wobrock et al., 2013; Yucel et al., 2012). A recent study in the EU-GEI sample using the degraded facial affect recognition task reported better facial emotion recognition processing in cannabis users with schizophrenia, their siblings, and healthy controls, both for total scores and for specific emotion recognition (neutral, happy, fearful, and angry) relative to non-users (Fusar-Poli et al., 2022b). However, other studies have observed poorer cognitive performance (D'Souza et al., 2005; Mata et al., 2008) or no differences in some cognitive tasks in people with schizophrenia using cannabis (Ahuir et al., 2021; Jockers-Scherubl et al., 2007; Sevy et al., 2007).

As social skills may be necessary for patients to acquire and use some substances, especially in context where access is more restricted, some researchers suggest that cannabis use in people with FEP could correlate with better social abilities, more drug taking opportunities, and better neurocognitive functioning (Arnold et al., 2015; Menendez-Miranda et al., 2019; Rodriguez-Sanchez et al., 2010; Yucel et al., 2012). Indeed, previous research in the EU-GEI sample suggests that better premorbid social and cognitive functioning could contribute to the likelihood of beginning to use cannabis before psychosis onset in patients with FEP (Ferraro et al., 2020).

In this study, we sought to examine for the first time the relationship between cannabis use and speech illusions, facial recognition processing deficits, and jumping to conclusions in patients with FEP, their non-psychotic siblings, and controls. These biases may underlie processes in social cognitive deficits in schizophrenia related with positive symptoms of schizophrenia such as persecutory delusions and auditory hallucinations. Following previous studies, we hypothesized that lifetime cannabis use would correlate with lower odds of cognitive biases relative to lifetime abstinence. As secondary objectives, we aimed to explore the association of frequency of cannabis use and cannabis potency with the odds of cognitive biases in cannabis users.

## Methods

### Study design

Members of a large, international, multisite, observational study, *The European Network of National Schizophrenia Networks Studying Gene-Environment Interactions* (EU-GEI), recruited participants between May 2010 and April 2015 from 17 catchment areas in 6 countries (Brazil, France, Italy, the Netherlands, Spain, and the United Kingdom). Study goals include analyzing the effects of genetic, environmental, and clinical variables and their interaction on the development, severity, and outcome of schizophrenia and other psychotic disorders. The EU-GEI team recruited a subset of people with FEP for a concurrent case-control study and controls from the same catchment areas. Additionally, they recruited patients' siblings to focus on the role of gene-environment interaction of the vulnerability and severity of psychosis in a family-based setting. Detailed study procedures are available in (Gayer-Anderson et al., 2020; Jongsma et al., 2018).

### Participants

The EU-GEI study sample included 1130 participants with FEP who attended mental health services in the catchment areas. Inclusion criteria for patients were: (1) diagnosis of non-organic psychotic disorder, (2) 18–64 years of age, (3) resident within one of the study catchment areas at the time of their first presentation. Exclusion criteria included: (1) presence of psychotic symptoms due to acute intoxication (ICD10: F1X.5) or organic psychosis (ICD10: F09), (2) previous contact with mental health services because of psychotic symptoms outside of the study period.

We also recruited siblings of FEP participants, aged 18 years or older (*N* = 265) via communications with the patient and/or his or her reference clinician. We excluded siblings if they presented current or past psychotic disorders (including a psychosis diagnosis within the time frame of the study) or if they had received treatment with antipsychotic medication.

We recruited volunteers from the same catchment areas and same age range as patients for the control sample by using a mixture of random and quota sampling to maximize the representativeness of samples in each catchment area. A total of 1497 controls agreed to participate in the study. We excluded controls if they had received psychotic disorder diagnoses, including during the study, or had undergone treatment with antipsychotic medication. More information about the recruitment procedures is available elsewhere (Di Forti et al., 2019; Gayer-Anderson et al., 2020; van Os et al., 2014).

For the purposes of this study, we included participants that had complete data on cannabis use and complete assessments of each of the three cognitive biases. This yielded a final sample size of 543 participants with FEP, 203 siblings, and 1168 controls (see online Supplementary Figure 1).

Local ethical committees of all sites approved the study at all the study sites. All participants gave written informed consent before entering the study. The authors assert that all procedures contributing to this work comply with the ethical standards of the relevant national and institutional committees on human experimentation and with the Helsinki Declaration of 1975, as revised in 2008.

### Measures

#### Demographic and clinical measures

We used the modified version of the Medical Research Council (MRC) socio-demographic scale to collect socio-demographic data (Mallett, Leff, Bhugra, Pang, & Zhao, 2002). Diagnoses were operationalized through the 90-item computerized Operational CRITeria (OPCRIT) system for psychosis (McGuffin, Farmer, & Harvey, 1991; Williams, Farmer, Ackenheil, Kaufmann, & McGuffin, 1996) in the group of patients. To estimate full scale-IQ scores (including Digit Symbol Substitution, Arithmetic, BlockDesign, and Information subtests) we used an abbreviated and adapted version of the WAIS (Velthorst et al., 2013). We measured clinical symptoms with the CAPE scale (Konings, Bak, Hanssen, van Os, & Krabbendam, 2006; Mossaheb et al., 2012), a questionnaire designed to rate self-reports of positive, negative, and depressive psychotic experiences. Duration of untreated psychosis (in weeks) was estimated with the Notting-ham Onset Schedule (Singh et al., 2005).

#### Measures of cannabis use

Information about cannabis use was collected with the Cannabis Experience Questionnaire (CEQ) further modified for the EUGEI study (CEQ_EU−GEI_) (Di Forti et al., 2019). The CEQ was modified to (1) include questions to assess dependence for cannabis use and other drugs, and (2) to describe use and changes in cannabis use over specific age periods. We asked participants whether they had ever used cannabis in their lifetime and, if so, we asked about the frequency, type (high or low potency), and duration of use based on the current pattern of use, or, when there was no current use of cannabis, based on the pattern of use that described best the overall pattern of cannabis use. We subdivided cannabis use frequency into three levels: daily use, occasional use (ranging from only once or twice in a lifetime to more than once a week), and never use (absence of lifetime cannabis use) (Di Forti et al., 2019; Ferraro et al., 2020).

We used data on the concentration of Δ⁹-tetrahydrocannabinol (THC) in the different types of cannabis available across Europe from in the European Monitoring Centre for Drugs and Drug Addiction 2016 report to create a measure of cannabis potency. A cutoff of THC = 10% was used to define the potency variable [high (> = 10%) v. low (<10%) potency] based on the mean THC concentration expected in the different types of cannabis available across the sites (for further information, please see online Supplementary Methods and Di Forti et al. (2019)).

#### Cognitive bias assessment

To explore speech illusions and aberrance salience we used the White Noise Task. The task consists of a random presentation of 75 audio fragments, 25 of which include just white noise, 25 that contain white noise and barely audible speech, and 25 that include white noise mixed with clearly audible speech, with positive, negative, or neutral affective content. Participants listened to the sounds binaurally through headphones. The length of the task was approximately 15 min. After each fragment, we asked participants to press specific buttons on a keyboard which reflected what they heard: (1) speech with positive content; (2) speech with negative content; (3) speech with neutral content; (4) absence of speech, and (5) presence of speech but uncertain to choose between positive, negative, or neutral emotional valence. Speech illusion was defined as a white noise fragment in which any speech was heard (option 1, 2, 3, or 5 in just white noise fragments). Only 25 out of 75 fragments contained white noises, so the maximum score for speech illusion was 25. A dichotomous variable was then calculated based on a cut-off of two or more speech illusions independently of the emotional valence attributed by the participant (Catalan et al., 2014).

We used the Benton Facial Recognition Test (BFRT) (Benton, 1994; Benton and Van Allen, 1968) to assess facial recognition processing. The BFRT is a face discrimination test in which participants are required to match a target face to either one face keeping the same viewpoint and lighting conditions (6 items) or three or six faces presented simultaneously that vary in viewpoints and lighting (16 items). The total score is calculated based on the total correct answers given (maximum score is 54). Deficits in facial recognition processing were defined as scores of 20 or fewer correct answers in the BFRT (Duchaine & Nakayama, 2006).

The Drawing to Decisions (DTD) index (Garety et al., 2015; So et al., 2016) was used to assess Jumping To Conclusion (JTC) bias. We obtained this index from the probabilistic reasoning (beads) task, with 60:40 task ratios. Two jars of beads were shown to participants in equal but opposite ratios (85 red and 15 blue and vice versa). Both jars were hidden, and researchers told participants that individual beads were drawn consecutively from one jar. The beads were actually presented in a prespecified sequence. Participants were required to either decide from which jar the beads had come or postpone the decision (up to a maximum of 20 beads). The key outcome variable employed as an index of the JTC bias was the number of ‘Draws-To-Decision’ (DTD); the lower the DTD, the greater the JTC bias. For the purposes of this study JTC bias was defined as a DTD index of 2 or less in the beads task (Klein & Pinkham, 2018), as participants are considered to have an extreme JTC bias when a decision is made after presentation of two or fewer items (Garety et al., 2005).

### Statistical analysis

We calculated means and standard deviations for continuous variables and frequency and percentages for categorical variables. For the comparisons in cognitive biases and frequency of cannabis use between FEP patients, siblings, and controls, we used Chi-square tests. We used ANOVA analyses to compare quantitative socio-demographic variables between the three groups. We used the Bonferroni test for post-hoc pairwise comparisons.

Considering the uneven distribution of the cognitive biases, we decided on a dichotomous analysis and used cutoffs based on previous studies using customary definitions of cognitive biases from the literature. To examine the influence of group and frequency of cannabis use on cognitive biases variables we conducted three sets of stratified logistic regression model analyses, as we were interested in analyzing the effect of cannabis use on the three cognitive biases separately. We adjusted our analyses for demographic variables: sex, age, years in education, ethnicity, employment (yes/no), migrant status (yes/no), and intelligence quotient (IQ). Variables were included in two models; Model 1 included clinical group (FEP, sibling, or control), demographic variables (sex, age, years of education, employment, and migration), and IQ; Model 2 added the frequency of lifetime cannabis use (never/occasional/daily) and the interaction between the frequency and the clinical group. We performed secondary analyses to examine the effects of frequency of use (occasional v. daily) and potency of cannabis (high v. low) in the group of cannabis users only.

We conducted supplementary analyses in the FEP group to additionally adjust Model 2 for the (i) severity of negative psychotic symptomatology (CAPE Negative) and (ii) Duration of Untreated Psychosis (DUP).

The level of statistical significance was set at *p* < 0.05. All statistical analyses were conducted using SPSS 25.

## Results

### Demographic and clinical characteristics

Socio-demographic characteristics of the sample are described in Table 1. We observed significant differences between the three clinical groups in sex, age, country, ethnicity, years in education, estimated IQ, and frequency of cannabis use. The FEP sample not analyzed in this study (*n* = 587; see online Supplementary Figure 1) showed significantly lower IQ, included lower rates of white and mixed ethnic groups and higher rates of black and north African ethnic groups and migrants, and higher rates of high-potency cannabis use relative to the FEP sample analyzed in the study (*n* = 543). Relative to the sibling and control samples not analyzed in the study, the sibling sample not analyzed in the study was significantly older and had fewer years in education and the control sample not analyzed in the study was older and included higher rates of white ethnic group. We did not find any significant differences in any other demographic or cannabis use variables on cognitive bias variables between the samples analyzed and not analyzed in this study.

**Table 1.** Demographic and clinical characteristics of the sample: patients with first-episode psychosis, siblings, and controls

Variables	FEP *N* = 543	Siblings *N* = 203	Controls *N* = 1168	Test (df)	*p*
Gender					
Male, *N* (%)	333 (61.3)	68 (47.6)	547 (46.8)	χ^2^ (2) = 54.5	<0.001
Female, *N* (%)	210 (38.7)	135 (66.5)	621 (53.2)		
Age, mean (s.d.)	31.5 (11)	30.7 (9.4)	35.7 (12.9)	F (2, 1911) = 31.6	<0.001
Country					
UK, *N* (%)	27 (5)	4 (2)	285 (24.4)	χ^2^ (10) = 196.4	<0.001
The Netherlands, *N* (%)	111 (20.4)	53 (26.1)	175 (15)		
Spain, *N* (%)	119 (21.9)	54 (26.6)	168 (14.4)		
France, *N* (%)	38 (7)	5 (2.5)	112 (9.6)		
Italy, *N* (%)	74 (13.6)	6 (3)	142 (12.2)		
Brazil, *N* (%)	174 (32)	81 (39.9)	286 (24.5)		
Ethnicity					
White, *N* (%)	372 (68.5)	153 (75.4)	905 (77.5)	χ^2^ (10) = 28.5	<0.001
Black, *N* (%)	50 (9.2)	13 (6.4)	96 (8.2)		
Mixed, *N* (%)	78 (14.4)	31 (15.3)	104 (8.9)		
Asian, *N* (%)	15 (2.8)	1 (0.5)	27 (2.3)		
North African, *N* (%)	16 (2.9)	4 (2)	19 (1.6)		
Other, *N* (%)	12 (2.2)	1 (0.5)	17 (1.5)		
Years education, mean (s.d.)	12.7 (4.4)	13.7 (4.6)	14.8 (4.2)	F (2, 1898) = 44.5	<0.001
Occupational status					
Ever employed: Yes, *N* (%)	493 (90.8)	190 (93.6)	1093 (93.6)	χ^2^ (2) = 4.524	0.104
Ever employed: No, *N* (%)	50 (9.2)	13 (6.4)	75 (6.4)		
Frequency of cannabis use					
Never, *N* (%)	211(38.9)	121 (59.6)	611 (52.3)	χ^2^ (4) = 176.6	<0.001
Occasional use, *N* (%)	173 (31.9)	70 (34.5)	476 (40.8)		
Daily use, *N* (%)	159 (29.3)	12 (5.9)	81 (6.9)		
Migrant					
Yes, *N* (%)	110 (20.3)	29 (14.3)	232 (19.9)	χ^2^ (2) = 3.814	0.149
No, *N* (%)	433 (79.7)	174 (85.7)	936 (80.1)		
IQ, mean (s.d.) CAPE negative, mean (s.d.) DUP in weeks, mean (s.d.)^a^	87.6 (17.7) 27.1 (7.9) 27.2 (55.1)	96.3 (14.7) 21.3 (6.1) −	103.6 (17.6) 21.2 (5.1) −	F (2) = 148.4 F (2) = 180.17 −	<0.001 <0.001 −

CAPE, Community Assessment of Psychic Experiences scale; DUP, Duration of Untreated Psychosis; FEP, First-Episode Psychosis; IQ, Intelligence Quotient.

a Outlier cases for DUP were not included in the analysis (*n* = 14).

### Cannabis use

Participants with FEP were less likely to have never used cannabis (*v.* occasional and daily use) than siblings and controls. Thus, 38.9% of FEPs, 59.6% of siblings, and 52.3% of controls had never used cannabis (*χ^2^*_(4)_ = 176.6 < 0.001). Patients were also more likely to have used cannabis daily (29.3%) relative to controls (6.9%) and siblings (5.9%); (*χ^2^*_(2)_ = 176.6; *p* < 0.001). Occasional use of cannabis was more frequent in the control (40.8%) than in the FEP group (31.9%); (*χ^2^*_(4)_ = 176.6 < 0.001) (see Table 1). In the subgroup of cannabis users, high potency (64%) was more frequent than low potency use (36%) in siblings (*χ^2^*_(2)_ = 8.248; *p* < 0.016), with no significant differences in the FEP or control groups.

### Association of clinical group and frequency of cannabis use with cognitive biases

FEP patients presented a higher proportion of all cognitive biases than siblings and controls. Across the whole sample, cognitive biases in the three tests were significantly more frequent in participants that had never used cannabis relative to those with occasional or daily use. Occasional users showed cognitive biases less frequently than daily users (all *p* < 0.001) (Table 2 and online Supplementary Figure 1).

**Table 2.** Percentage of participants with cognitive biases (speech illusions, facial recognition processing *deficit* (*BFR*), *and jumping to cbias* (*DTD*) *according to clinical group and cannabis use*

	Group				Cannabis use			
	FEP	Siblings	Controls	χ^2^; p	Daily	Occasional	Never	χ^2^; *p*
*Speech illusion*	27.6	24.6	18.8	17.83;<0.001	21	14.7	27.7	39.99;<0.001
*BFR*	36.1	24.6	21.8	17.67;<0.001	24.2	20.6	31	23.32;<0.001
*DTD*	56.5	50.7	45.6	17.83;<0.001	48.8	39.2	57	51.91;<0.001

BFR, Benton facial recognition; DTD, drawing to decision.

Table 3 shows the logistic regression models of ***speech illusions* (*SI*)**. In model 1, clinical group did not show a significant association with the presence of SI. In model 2, we found that frequency of cannabis use was a predictive factor in the model, with no significant effects of clinical group or the interaction between clinical group and frequency of use. Specifically, occasional use of cannabis correlated with 0.64-fold decreased odds (OR = 0.646; 95% CI 0.457–0.9113), and daily use of cannabis with 0.60-fold decreased odds (OR = 0.605; 95% CI 0.368–0.997) of having SI relative to never use of cannabis. The resulting final model explained 16.5% of the variance of the presence of SI (*R*^2^ = 0.165, *p* < 0.001). Secondary analyses conducted in cannabis users showed a significant effect of low-potency cannabis on SI (OR = 1.829; 95%CI 1.297–2.578). Frequency of cannabis use (occasional v. daily) was no longer a predictive factor in the model and the effects decreased substantially. We found no significant interaction between clinical group and the frequency of cannabis use or potency (see online Supplementary Table 1).

**Table 3.** Logistic regression models for speech illusion

Speech illusion (yes)	*β*	Error	Sig.	OR [CI 95%]
**Model 1**		χ^2^_(13)_ = 18 122, *p* < 0. 001; *R*^2^ = 0.149		
Group (controls)			0.650	
FEP	0.121	0.150	0.420	1.129 [0.841–1.516]
Siblings	0.133	0.200	0.507	1.142 [0.771–1.692]
**Model 2**		χ^2^_(17)_ = 201 605; *p* < 0.001; *R*^2^ = 0.165		
Group (controls)			0.097	
FEP	0.259	0.218	0.236	1.295 [0.844–1.987]
Siblings	−0.705	0.456	0.122	0.494 [0.202–1.208]
Cannabis use (never)			**0.034**	
Occasional	−0.437	0.176	**0.013**	**0.646 [0.457–0.913]**
Daily	−0.502	0.254	**0.049**	**0.605 [0.368–0.997]**

*Note.* All the models are adjusted for sex, age, ethnicity, years in education, employment, migration, and estimated intelligence quotient (IQ). Age was significant in Model 1 (*p* < 0.001); ethnicity and education were significant in Models 1 and 2 (*p* < 0.001) (not shown in the Table).

Model 1 = Group + sex, age, ethnicity, years in education, employment, migrant; Model 2 = Model 1 + frequency of cannabis use + interaction group × frequency of cannabis use (*p* > 0.05 for all interactions studied, data not shown in the Table). Significant results are highlighted in bold.

Presence of aberrance salience was considered as presence of speech illusion (speech illusion/yes) using a cutoff of two or more speech illusions.

*R*^2=^Nagelkerke's *r*^2^; Hosmer–Lemeshow test *p* > 0.05 for Models 1 and 2.

CI, confidence interval; FEP, first-episode psychosis; OR, odds ratio.

Table 4 shows the logistic regression models for the ***BFR***. In model 1, the FEP group showed significantly higher odds of facial recognition processing (FRP) deficits compared with controls (OR = 1.467;95%CI 1.122–1.918), with no significant differences with their siblings. After controlling for frequency of cannabis in model 2, the difference in patients and controls remained significant, with comparable effects (OR = 1.642; 95%CI 1.123–2.402), while frequency of cannabis use was not a significant predictor in the model. The interaction effect between clinical group and frequency of cannabis use in the model was also not significant. The final model accounted for 12.1% of the explained variance (*R*^2^ = 0.121, *p* < 0.001). Secondary analyses including cannabis users only showed a comparable effect of clinical group (OR = 1.588; 95%CI 1.104–2.284, a significant effect of potency (OR = 1.393; 95%CI 1.031–1.882), and no significant effects of frequency of cannabis use or the interaction between clinical group and frequency of use or potency in the models (see online Supplementary Table 2).

**Table 4.** Logistic regression models for facial recognition processing (FRP) deficit

BFR (Low)	*β*	Error	Sig.	OR [CI 95%]
**Model 1**		χ^2^_(13)_ = 139.737; *p* < 0.001; *R*^2^ = 0.111		
Group (controls)			**0.018**	
FEP	0.383	0.137	**0.005**	**1.467 [1.122–1.918]**
Siblings	0.066	0.192	0.732	1.068 [0.733–1.556]
**Model 2**		χ^2^_(17)_ = 153.272; *p* < 0.001; *R*^2^ = 0.121		
Group (controls)			**0.003**	
FEP	0.496	0.194	**0.011**	**1.642 [1.123–2.402]**
Siblings	−0.656	0.397	0.099	0.519 [0.238–1.130]
Cannabis use (never)			0.157	
Occasional	−0.154	0.162	0.222	0.821 [0.598–1.127]
Daily	−0.442	0.237	0.056	0.637 [0.401–1.011]

*Note.* All the models are adjusted for sex, age, ethnicity, years in education, employment, migration, and estimated intelligence quotient (IQ). Age was significant in Model 1 (*p* < 0.001); ethnicity and education were significant in Models 1 and 2 (*p* < 0.001); IQ was significant in Model 1 (*p* < 0.001) and 2 (*p* = 0.002) (not shown in Table).

Model 1 = Group + sex, age, ethnicity, education, employment, migrant; Model 2 = Model 1 + Frequency of cannabis use + Interaction group x frequency of cannabis use (*p* > 0.05 in all interactions studied, data not shown in the Table). Significant results are highlighted in bold.

Deficits in FRP deficits were defined as scores of 20 or less in the BFR test (BFR low).

*R*^2^ = Nagelkerke's *r*^2^; Hosmer–Lemeshow test *p* > 0.05 for Models 1 and 2.

BFR, Benton facial recognition; CI, confidence interval; FEP, first-episode psychosis; OR, odds ratio.

Table 5 shows the logistic regression analyses for the **DTD.** We did not find a significant effect of clinical group on JTC bias in Model 1 or 2. After including frequency of use in Model 2, participants who used cannabis occasionally or daily showed significantly lower odds of JTC bias compared with abstainers in the whole sample (OR = 0.602; 95%CI 0.460–0.787) and (OR = 0.625; 95% CI 0.422–0.925), respectively. There was no significant interaction between clinical group and frequency of cannabis use in the model. This model explained 19.3% of the variance (R2 = 0.193, *p* < 0.001;).

**Table 5.** Logistic regression models for jumping to conclusions bias (drawing to decision index)

DTD (≤2 draws)	β	Error	Sig.	OR [CI 95%]
Model 1		χ^2^_(13)_ = 266.438; *p* < 0.001; *R*^2^ = 0.184		
Group (controls)			0.941	
FEP	0.032	0.128	0.802	1.033 [0.803–1.328]
Siblings	0.050	0.171	0.768	1.052 [0.753–1.469]
Model 2		χ^2^_(17)_ = 280.908; *p* < 0.001; *R*^2^ = 0.193		
Group (controls)			0.340	
FEP	0.232	0.173	0.179	1.261 [1.261 [0.899–1.769]
Siblings	0.230	0.262	0.379	1.259 [0.754–2.103]
Cannabis Use (Never)			**<0.001**	
Occasional	−0.508	0.137	**<0.001**	**0.602 [0.460–0.787]**
Daily	−0.471	0.201	**0.019**	**0.625 [0.422–0.925]**

*Note.* All the models are adjusted for sex, age, ethnicity, years in education, employment, migration, and estimated intelligence quotient (IQ). Age was significant in Model 1 (*p* < 0.001); ethnicity, education, and IQ were significant in Models 1 and 2 (*p* < 0.001) (not shown in the Table).

Model 1 = group + sex, age, ethnicity, education, employment, migrant; Model 2 = Model 1 + frequency of cannabis use + Interaction group × frequency of cannabis use (*p* > 0.05 for all the interactions studied, data not shown). Significant results are highlighted in bold.

Jumping to conclusions bias was defined as a DTD index of 2 or less draws in the beads task.

*R*^2=^Nagelkerke's *r*^2^; Hosmer–Lemeshow test *p* > 0.05 for Models 1 and 2.

CI, confidence interval; DTD, drawing to decision; FEP, first-episode psychosis; OR, odds ratio.

Secondary analyses in the subgroup of cannabis users showed low-potency as a significant predictor of DTD (OR = 1.661; 95%CI 1.271–2.171), with no significant effect of frequency of use (occasional v. daily) or the interaction between clinical group and frequency of use or potency in the model (see online Supplementary Table 3).

Supplementary analyses in the FEP sample adjusted for the duration of untreated psychosis or the score on the negative CAPE subscale found comparable effects for the frequency of cannabis use variables and similar predictive capacity to those from the main models (see online Supplementary Tables 4–6).

## Conclusions

To the best of our knowledge, this is the first study to examine the odds of speech illusions, facial recognition deficits, and jumping to conclusions biases in a large international sample of patients with FEP, their siblings, and healthy controls. We found that FEP patients showed greater likelihood of facial recognition processing (FRP) deficits than siblings and controls. Contrary to previous findings in schizophrenia (Hoffman, 1999; Hoffman et al., 2007; Holt et al., 2006; Kapur, 2003), we did not find significantly higher odds of speech illusions (SI) and JTC in FEP patients relative to siblings and controls, in this large international study.

Together with theory of mind, facial processing is critical for meaningful social interactions and for guiding social behavior and deficits in FRP may underlie some of the social cognition deficits in patients with psychosis (Comparelli et al., 2014; Green et al., 2012; Mucci et al., 2021; Velthorst et al., 2017). In this study, we found more frequent FRP deficits in individuals with FEP than in their siblings and controls, thus suggesting that FRP deficits could be a specific marker related to psychosis but not genetically shared with siblings. This is consistent with previous studies reporting increased likelihood of cognitive biases related to facial processing deficits in individuals with psychosis relative to their siblings or controls (Fusar-Poli et al., 2022b; Shin et al., 2008; Tripoli et al., 2022; van 't Wout et al., 2004) and with previous evidence in the EU-GEI sample. For example, Fusar-Poli et al. (2022a, 2022b) found that deficits in facial emotion recognition (measured by total Degraded Facial Affect Recognition task scores) were greater in individuals with schizophrenia than in siblings, who showed greater deficits than healthy controls. In our study, we did not find differences between siblings and controls regarding FRP deficits. Our divergent findings may be due to differing tasks or a more diagnostically heterogenous sample of FEP and their siblings in our study (Fusar-Poli et al., 2022b).

We did not find an increased likelihood of aberrant salience in FEP patients than in siblings or healthy controls. Previous studies found that proneness to aberrant salience correlated with familial vulnerability for psychosis, and was more likely found in delusional schizophrenia patients than in non-delusional and healthy subjects or their siblings, thus suggesting a familial liability to psychosis associated with aberrant salience (Catalan et al., 2014; Galdos et al., 2011; Hoffman, 1999; Hoffman et al., 2007; Holt et al., 2006; Schepers et al., 2019). Also, previous studies focusing on FEP patients showed higher rates of speech illusions than the control group, contrary to our findings, and speech illusions correlated with positive symptomatology (Catalan et al., 2014). Our results may differ in part because most of the studies mentioned did not include FEP-only samples or studied smaller samples with more unspecific tasks to measure these cognitive processes.

Contrary to our JTC findings, previous literature reports that patients with psychosis frequently use less information to arrive at a decision than controls and show increased risk of JTC bias (Ahuir et al., 2021; Dudley et al., 2016; Henquet et al., 2022; Hofer et al., 2010; Lee et al., 2013; Ross, McKay, Coltheart, & Langdon, 2015). Similarly, previous studies based on the EU-GEI WP6 sample including schizophrenia patients, their siblings and healthy controls, showed increased odds of JTC bias in both patients and siblings relative to controls, thus suggesting an association between JTC bias and familial risk for psychosis (Henquet et al., 2022). Our different results may be because some of these studies were conducted in chronic schizophrenia samples or did not include FEP patients only. Indeed, a previous study observed a higher prevalence of JTC bias in individuals with schizophrenia than in individuals with recent-onset psychosis. Inconsistent findings could also result from previous studies not including IQ as a potential confounding variable in the analyses. In fact, the FEP group showed increased odds of JCT bias relative to controls in our sample in the analyses not adjusted by IQ, with no significant effect of the clinical group after controlling for this variable.

Our overall results confirmed our hypothesis that lifetime abstention from cannabis correlated with the presence of SI and JTC bias compared to daily or occasional cannabis use, with a comparable effect across FEP patients, siblings, and controls. As social skills could enable FEP patients to acquire and maintain a drug habit, or facilitate the use of drugs in social environments (Menendez-Miranda et al., 2019), cannabis use in people with FEP could be indicative of better or preserved social abilities, more drug taking opportunities, better neurocognitive functioning, and therefore, fewer and less significant cognitive biases (Arnold et al., 2015; Rodriguez-Sanchez et al., 2010; Yucel et al., 2012). In this respect, a previous analysis of the EU-GEI sample observed better social adjustment prior to FEP in occasional and daily cannabis users relative to non-users. This difference was not observed in the control group (Ferraro et al., 2020). Previous literature on patients with psychosis has also reported premorbid differences between cannabis users and non-users, with cannabis users potentially developing psychosis at an earlier age through an alternative pathway with less cognitive vulnerability (both neurocognitive and social cognitive) (Myles, Myles, & Large, 2016; Schnakenberg Martin et al., 2016).

Taken together, we conclude that FEP cannabis users could constitute a group of less cognitively impaired individuals (Arnold et al., 2015) in whom cannabis use would be an important contributor to psychosis risk (Ferraro et al., 2020; Ferraro et al., 2019; Ferraro et al., 2013). In fact, we found no significant differences in cognitive biases between occasional and daily use in cannabis users, supporting the idea that FEP cannabis users and nonusers may represent distinct populations with differing premorbid load and risk factors, rather than there being a global direct protective effect of cannabis use on social cognition. Exploring if these associations exist both before and after the FEP warrants future longitudinal studies.

Among lifetime cannabis users, the use of low-potency cannabis was significantly associated with increased odds of SI, FRP, and JTC relative to high-potency cannabis use, with comparable effects across FEP patients, their siblings, and controls. Potent cannabis varieties, with high concentrations of delta-9-tetrahydrocannabinol (Δ9-THC), correlate with the most harm to mental health (Di Forti et al., 2015; Freeman et al., 2018) and recent studies based on the EU-GEI sample have shown how FEP patients with a history of daily use of high-potency cannabis present with more positive symptoms, compared with those who never used cannabis or used low potency variants (Quattrone et al., 2021). However, the relationship between the potency of cannabis and neurocognition is still unclear, and growing evidence suggests that two of the main cannabinoids, THC and CBD, display opposing neural, cognitive, and behavioral effects (Iseger & Bossong, 2015). Moreover, a study of cannabis users implicated THC in impaired facial emotional recognition, while CBD improved facial emotional recognition and attenuated THC-induced impairment (Hindocha et al., 2015). Our results show that FEP patients who use high potency variants of cannabis show less cognitive bias than those who use low potency cannabis, contrary to previous findings. One possible explanation could be related with the gateway hypothesis; FEP patients who smoke low potency variants of cannabis and show social cognitive impairment may not progress to later consumption of higher potency variants of cannabis.

This study has several limitations. First, cross-sectional studies limit analyses to a descriptive level and we could not assess causality. We also lacked a direct measure of social skills. Future studies using methodologies that allow for a more in-depth analysis and using a prospective design could clarify the longitudinal association between cannabis use and the risk of cognitive biases, as well as ascertain the specific role of social skills in this association. Second, cannabis use was not corroborated with the collection of biological samples. Instead, we used self-reporting tools, with a risk of recall bias and we did not conduct specific memory assessments. Notwithstanding, previous studies reported that the use of interviews to collect information about drug use in the adult population is generally reliable and valid (Curran et al., 2019; Freeman et al., 2014; Van Dorn, Desmarais, Scott Young, Sellers, & Swartz, 2012). Third, although we analyzed a large sample of participants with FEP, their siblings, and healthy controls, our findings may not be generalizable to all FEP and sibling populations. Exclusion of participants from the analysis due to lacking information on cannabis use or cognitive bias variables could influence the representativeness of our findings. Fourth, in keeping with other EU-GEI publications we classified cannabis use frequency in three categories (never use, occasional use, daily use). However, the heterogeneity in the “occasional use” category may have influenced our results. Finally, despite potential effect of antipsychotic treatments and other interventions on cognitive functioning in first-episode psychosis (Allot et al., 2023), we did not consider pharmacological treatments and other cognitive or psychosocial interventions in our analyses. Future studies should assess the effect of these factors on the association between cannabis use and cognitive biases.

In conclusion, FRP deficits were more prevalent in FEP patients than in their siblings or control participants, with no significant differences between the three groups in other cognitive biases such as speech illusions or JTC bias in the adjusted analyses. Cognitive biases were more frequent in cannabis abstainers and in participants who used low potency of cannabis relative to daily and occasional users and high potency participants respectively. Our findings suggest that FEP patients who have never used cannabis on a regular basis would be more likely to present cognitive biases. Considering the detrimental effects of cannabis on psychosis risk even beyond genetic predisposition (Ferraro et al., 2023), future studies should elucidate this association and its potential clinical implications further.

## Supporting information

Roldan et al. supplementary material Roldan et al. supplementary material

## Supplementary material

The supplementary material for this article can be found at https://doi.org/10.1017/S0033291724001715.
