# Supplementary material for: Cannabis use and cognitive biases in people with first-episode psychosis and their siblings
Source: Psychol Med. 2024 Nov 22;54(15):4095–105. doi: 10.1017/S0033291724001715 (PMC11650177; doi:10.1017/S0033291724001715)
Supplement: Roldan et al. supplementary material [file S0033291724001715sup001.docx]

**SUPPLEMENTARY MATERIAL**

**Supplementary Methods**

**Measures of cannabis use: potency**

Participants were instructed to identify, in their native language, the type of cannabis they primarily used during their period of consumption, by responding to the question: "What type of cannabis did you mostly use?" The potency classification was established using a THC threshold of 10%, derived from the average THC concentrations expected in various types of cannabis available across the study sites, as documented by the European Monitoring Centre for Drugs and Drug Addiction (EMCDDA, 2016), and supported by national data on cannabis potency. The low-potency cannabis group (THC < 10%) encompassed hash/resin from the UK and Italy, imported herbal cannabis from the UK, Italy, Spain, and France, Brazilian marijuana and hash, as well as Dutch Geimporteerde Wiet. The high-potency category (THC ≥ 10%) included all other types mentioned by participants using their local street names, such as UK home-grown skunk/sensimilla, UK Super Skunk, Italian home-grown skunk/sensimilla, Italian Super Skunk, Dutch Nederwiet, Nederhasj, and geimporteerde hasj, Spanish and French Hashish (from Morocco), Spanish home-grown sensimilla, French home-grown skunk/sensimilla/super-skunk, and Brazilian skunk. Additional details can be found in Di Forti et al. (2019).

**Supplementary Methods**

*Supplementary Table 1. Logistic Regression models for Speech Illusion including participants with cannabis use only (n=1,155)*

| **Speech Illusion (Yes)** | β | Error | Sig. | OR, [95% CI] |
| --- | --- | --- | --- | --- |
| **Model 2** |  | χ^2^_(15)_ = 92.599; p < 0.001 | | |
| Group (controls) |  |  | 0.113 |  |
| FEP | 0.335 | 0.212 | 0.113 | 1.398 [0.923-2.118] |
| Siblings | -0.424 | 0.437 | 0.332 | 0.655 [0.278-1.542] |
| Frequency of Use (Occasionally) | 0.101 | 0.192 | 0.598 | 1.107 [0.706-1.611] |
| Potency of cannabis (Low) | 0.604 | 0.175 | **<0.001** | **1.829 [1.297-2.578]** |

*Note.* Model 2 (*R^2^* = 0.130) was adjusted for gender, age, ethnicity, years in education, employment, migration, estimated intelligence quotient (IQ), frequency of cannabis use (occasional or daily use) potency of use (low or high potency) and interaction group x frequency and group x potency (p >0.05 for all interactions studied, data not shown in the Table). Educational level was significant in the model (p < 0.001) (not shown in the Table). Hosmer-Lemeshow test p > 0.05. Significant results are highlighted in bold.

Presence of aberrant salience was considered as presence of Speech illusion (Speech Illusion/ Yes) using a cut-off of two or more speech illusions.

Abbreviations: CI= Confidence Interval; FEP= First-Episode Psychosis, OR = Odds Ratio

*Supplementary Table 2. Logistic Regression models for Facial Recognition Processing (FRP) deficit including participants with cannabis use only (n=1,171)*

| **BFR (Low)** | β | Error | Sig. | OR, [95% CI] |
| --- | --- | --- | --- | --- |
| **Model 2** |  | χ^2^_(15)_ = 76.023; p < 0.001 | | |
| Group (controls) |  |  | **0.008** |  |
| FEP | 0.463 | 0.185 | **0.013** | **1.588 [1.104-2.284]** |
| Siblings | -0.477 | 0.379 | 0.621 | 0.295 [0.295-1.306] |
| Cannabis Use (Occasionally) | -0.239 | 0.175 | 0.172 | 0.787 [0.558-1.10] |
| Potency of cannabis (Low) | 0.332 | 0.153 | **0.031** | **1.393 [1.031-1.882]** |

*Note.* Model 2 (*R^2^* = 0.089) was adjusted for gender, age, ethnicity, years in education, employment, migration, estimated intelligence quotient (IQ), frequency of cannabis use (occasional or daily use), potency of use (low or high potency) and interaction group x frequency and group x potency (p >0.05 for all interactions studied, data not shown in the Table). Years in education (p<0.001) and estimated IQ (p = 0.001) were significant in the model (not shown in the Table). Hosmer-Lemeshow test p > 0.05. Significant results are highlighted in bold.

Deficits in Facial Recognition Processing were defined as scores of 20 or less in the BFR test (BFR Low).

Abbreviations: BFR= Benton Facial Recognition, CI= Confidence Interval; FEP= First-Episode Psychosis, OR = Odds Ratio.

*Supplementary Table 3. Logistic Regression models for Jumping to Conclusion bias (Drawing to Decision index) including participants with cannabis use only (n=1,160)*

| **DTD (≤2 draws)** | β | Error | Sig. | OR, [95% CI] |
| --- | --- | --- | --- | --- |
| **Model 2** |  | χ^2^_(15)_ = 213.233; p < 0.001 | | |
| Group (controls) |  |  | 0.210 |  |
| FEP | 0.272 | 0.164 | 0.097 | 1.312 [0.952-1.809] |
| Siblings | 0.267 | 0.265 | 0.314 | 1.306 [0.777-2.195] |
| Cannabis Use (Occasionally) | 0.012 | 0.155 | 0.936 | 1.013 [0.747-1.373] |
| Potency of cannabis (Low) | 0.507 | 0.137 | **<0.001** | **1.661 [1.271-2.171]** |

*Note.* Model 2 (*R^2^* = 0.082) was adjusted for gender, age, ethnicity, educational level, employment, migration, estimated intelligence quotient (IQ), frequency of cannabis use (occasional or daily use) potency of use (low or high potency) and interaction group x frequency and group x potency (p >0.05 for all interactions studied, data not shown in the Table). Age (p <0.001), ethnicity (p= 0.006), estimated IQ (p<0.001), and years in education p< 0.001) were significant in the model (not shown in the Table). Hosmer-Lemeshow test p > 0.05. Significant results are highlighted in bold.

Jumping to conclusions bias was defined as a DTD index of 2 or less draws in the beads task.

Abbreviations: DTD= Drawing to Decision, CI= Confidence Interval; FEP= First-Episode Psychosis, OR = Odds Ratio.

*Supplementary Table 4. Logistic Regression models for Speech Illusion including participants with first-episode psychosis only (n=517)*

| **Speech Illusion (Yes)** | β | Error | Sig. | OR, [95% CI] |
| --- | --- | --- | --- | --- |
| **Model 1** |  | χ^2^_(13)_ = 66.760; p < 0.001; R2 = 0.191 | | |
| Frequency of Use (Never)  Occasionally  Daily | -0.488  -0.532 | 0.290  0.318 | 0.152  0.93  0.94 | 0.614 [0.348-1.084]  0.587 [0.315-1.095] |
| **Model 2** χ^2^_(14)_ = 67.230; p < 0.001; R2 = 0.193 | | | | |
| Frequency of Use (Never)  Occasionally  Daily  CAPE_Neg | -0.493  -0.536  0.010 | 0.291  0.318  0.014 | 0.149  0.90  0.92  0.492 | 0.611 [0.345-1.080]  0.585 [0.314-1.092]  1.010 [0.982-1.038] |
| **Model 3** χ^2^_(15)_ = 67.275; p < 0.001; R^2^ = 0.195 | | | | |
| Frequency of Use (Never)  Occasionally  Daily  DUP (weeks) | -0.529  -0.590  0.002 | 0.294  0.319  0.002 | 0.120  0.071  0.079  0.423 | 0.589 [0.331-1.047]  0.571 [0.306-1.067]  1.002 [0.998-1.006] |

*Note.* Model 1 was adjusted for gender, age, ethnicity, years in education, employment, migration, estimated intelligence quotient (IQ), frequency of cannabis use (never, occasional or daily use). Educational level was significant in the model (p < 0.001) (not shown in the Table). Model 2 = Model 1 + CAPE Negative. Model 3 = Model 1 + Duration of untreated psychosis (DUP). Outlier cases for DUP were not included in the analysis (n = 14). Hosmer-Lemeshow test p > 0.05. Significant results are highlighted in bold.

Presence of aberrance salience was considered as presence of speech illusion (Speech Illusion/ Yes) using a cut-off of two or more speech illusions.

Abbreviations: CI= Confidence Interval; FEP= First-Episode Psychosis, OR = Odds Ratio

*Supplementary Table 5. Logistic Regression models for Facial Recognition Processing (FRP) including participants with first-episode psychosis only (n=517)*

| **BFR (Low)** | β | Error | Sig. | OR, [95% CI] |
| --- | --- | --- | --- | --- |
| **Model 1** |  | χ^2^_(13)_ = 55.290 ; p < 0.001; R2 =0.152 | | |
| Frequency of Use (Never)  Occasionally  Daily | -0.085  -0.506 | 0.260  0.296 | 0.190  0.744  0.087 | 0.919 [0.552-1.528]  0.603 [0.338-1.076] |
| **Model 2** χ^2^_(14)_ = 57.959; p < 0.001; R2 =0.159 | | | | |
| Frequency of Use (Never)  Occasionally  Daily  CAPE_Neg | -0.095  -0.522  0.021 | 0.261  0.297  0.013 | 0.176  0.716  0.079  0.103 | 0.909 [0.545-1516]  0.593 [0.331-1.632]  1.021 [0.996-1.048] |
| **Model 3** χ^2^_(14)_ = 54.695; p < 0.001; R^2^ = 0.153 | | | | |
| Frequency of Use (Never)  Occasionally  Daily  DUP (weeks) | -0.095  -0.530  0.001 | 0.263  0.297  0.002 | 0.168  0.718  0.075  0.565 | 0.909 [0.544-1.522]  0.589 [0.329-1.054]  1.001 [0.997-1.005] |

*Note.* Model 1 was adjusted for gender, age, ethnicity, years in education, employment, migration, estimated intelligence quotient (IQ), frequency of cannabis use (never, occasional or daily use). Educational level was significant in the model (p < 0.001) (not shown in the Table). Model 2 = Model 1 + CAPE Negative. Model 3 = Model 1 + Duration of untreated psychosis (DUP). Outlier cases for DUP were not included in the analysis (n = 14). Hosmer-Lemeshow test p > 0.05. Significant results are highlighted in bold.

Deficits in Facial Recognition Processing were defined as scores of 20 or less in the BFR test (BFR Low).

Abbreviations: BFR= Benton Facial Recognition, CI= Confidence Interval; FEP= First-Episode Psychosis, OR = Odds Ratio.

*Supplementary Table 6. Logistic Regression models for Jumping to Conclusion bias (Drawing to Decision index) including participants with first-episode psychosis only (n=1,155)*

| **DTD (≤2 draws)** | β | Error | Sig. | OR, [95% CI] |
| --- | --- | --- | --- | --- |
| **Model 1** |  | χ^2^_(13)_ = 95.187; p < 0.001; R2 = 0.242 | | |
| Frequency of Use (Never)  Occasionally  Daily | -0.106  -0.183 | 0.258  0.284 | 0.811  0.682  0.521 | 0.900 [0.543-1.491]  0.833 [0.477-1.454] |
| **Model 2** χ^2^_(14)_ = 96.608; p < 0.001; R2 = 0.245 | | | | |
| Frequency of Use (Never)  Occasionally  Daily  CAPE_Neg | -0.093  -0.164  -0.016 | 0.258  0.285  0.230 | 0.846  0.717  0.565  0.233 | 0.911 [0.549-1.511]  0.849 [0.485-1.485]  0.985 [0.960-1.010] |
| **Model 3** χ^2^_(14)_ = 91.921; p < 0.001; R^2^ = 0.238 | | | | |
| Frequency of Use (Never)  Occasionally  Daily  DUP (weeks) | -0.078  -0.152  -0.001 | 0.260  0.286  0.002 | 0.868  0.765  0.595  0.697 | 0.925 [0.556-1.541]  0.859 [0.491-1.504]  0.999 [0.996-1.003] |

*Note.* Model 1 was adjusted for gender, age, ethnicity, years in education, employment, migration, estimated intelligence quotient (IQ), frequency of cannabis use (never, occasional or daily use). Educational level was significant in the model (p < 0.001) (not shown in the Table). Model 2 = Model 1 + CAPE Negative. Model 3 = Model 1 + Duration of untreated psychosis (DUP). Outlier cases for DUP were not included in the analysis (n = 14). Hosmer-Lemeshow test p > 0.05. Significant results are highlighted in bold.

Jumping to conclusions bias was defined as a DTD index of 2 or less draws in the beads task.

Abbreviations: CI=Confidence Interval; DTD= Drawing to Decision, FEP= First-Episode Psychosis, OR = Odds Ratio.

*Supplementary Figure 1. Flow chart of the study sample*

**2892 total study sample**

**1130 FEP**

**1497 healthy controls**

**Missing data of cannabis use = 48**

**Missing data of DTD= 228; BFR =237; Speech Illusion= 266**

**Total missing data because of CN and/or DTD, BFR, and speech illusion= 587**

**543 FEP**

**332 cannabis users**

**211 cannabis non-users**

**557 cannabis users**

**611 cannabis non-users**

**1168 healthy controls**

**265 siblings**

**Missing data of cannabis use = 15**

**Missing data of DTD= 15; BFR =17; Speech Illusion= 14**

**Total missing data because of CN and/or DTD, BFR and speech illusion= 62**

**Missing data of cannabis use = 17**

**Missing data of DTD= 165; BFR =148; Speech Illusion= 165**

**Total missing data because of CN and/or DTD, BFR and speech illusion= 329**

**203 siblings**

**82 cannabis users**

**121 cannabis non-users**

*Note*: *DTD= Drawing to

**Decision Index; BFR= Benton Recognition Test; CN= Cannabis; FEP= First Episode of Psychosis**

*Supplementary Figure 2*: *Percentage of participants showing cognitive biases according to clinical group and frequency of cannabis use and (Patients with First-Episode Psychosis (FEP), Siblings, and Controls)*


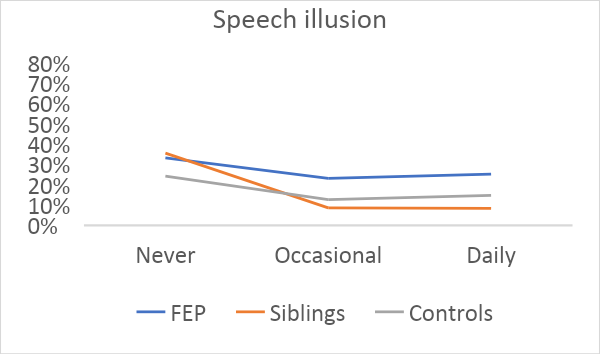

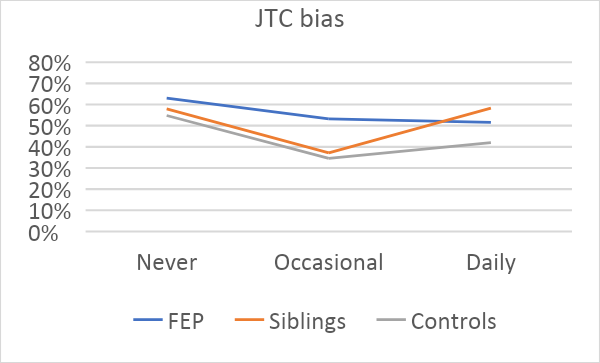


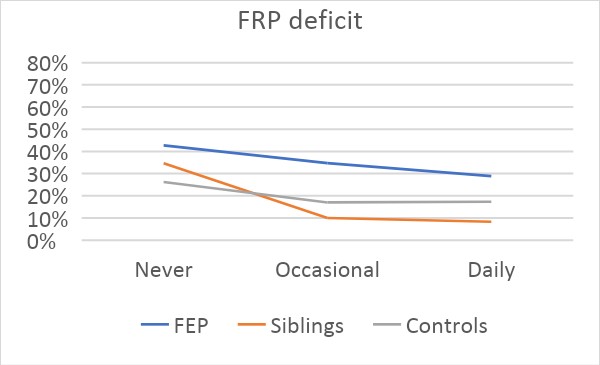


*Note*. All the figures represent the percentage of participants with cognitive biases in each clinical group according to the frequency of cannabis use.

Abbreviations: FRP= Face Recognition Processing; JTC= Jumping To Conclusions.

**Supplementary References**

Di Forti, M., Quattrone, D., Freeman, T. P., Tripoli, G., Gayer-Anderson, C., Quigley, H., . . . Group, E.-G. W. (2019). The contribution of cannabis use to variation in the incidence of psychotic disorder across Europe (EU-GEI): a multicentre case-control study. *Lancet Psychiatry, 6*(5), 427-436. doi:10.1016/S2215-0366(19)30048-3

EMCDDA, E. M. C. f. D. a. D. A. (2016). *European Drug Report 2016: Trends and Developments*. Luxembourg: Publications Office of the European Union.
